# Phase IIb randomized trial of adjunct immunotherapy in patients with first-diagnosed tuberculosis, relapsed and multi-drug-resistant (MDR) TB

**DOI:** 10.1186/1476-8518-9-3

**Published:** 2011-01-18

**Authors:** Dmitry A Butov, Yuri N Pashkov, Anna L Stepanenko, Aleksandra I Choporova, Tanya S Butova, Dendev Batdelger, Vichai Jirathitikal, Aldar S Bourinbaiar, Svetlana I Zaitzeva

**Affiliations:** 1 Department of Phtysiatry and Pulmonology, Kharkov National Medical University; Kharkov, Ukraine; 2 National Research Center for Infectious Diseases (NRCID), Ulaanbaatar, Mongolia; 3 Immunitor Thailand Co., LLC, Bangpakong Industrial Park, Chachoengsao, Thailand; 4 Immunitor USA Inc., College Park, MD 20740, USA

## Abstract

Placebo-controlled, randomized, phase 2b trial was conducted in 34 adults comprising 18 first-diagnosed (52.9%), 6 relapsed (17.6%), and 10 MDR-TB (29.4%) cases to investigate the safety and efficacy of an oral immune adjunct (V5). The immunotherapy (N = 24) and placebo (N = 10) arms received once-daily tablet of V5 or placebo for one month in addition to conventional anti-TB therapy (ATT) administered under directly observed therapy (DOT).

The enlarged liver, total bilirubin, erythrocyte sedimentation rate, lymphocyte and leukocyte counts improved significantly in V5 recipients (P = 0.002; 0.03; 8.3E-007; 2.8E-005; and 0.002) but remained statistically unchanged in the placebo group (P = 0.68; 0.96; 0.61; 0.91; and 0.43 respectively). The changes in hemoglobin and ALT levels in both treatment arms were not significant. The body weight increased in all V5-treated patients by an average 3.5 ± 1.8 kg (P = 2.3E-009), while 6 out of 10 patients on placebo gained mean 0.9 ± 0.9 kg (P = 0.01). Mycobacterial clearance in sputum smears was observed in 78.3% and 0% of patients on V5 and placebo (P = 0.009). The conversion rate in V5-receiving subjects with MDR-TB (87.5%) seemed to be higher than in first-diagnosed TB (61.5%) but the difference was not significant (P = 0.62). Scoring of sputum bacillary load (range 3-0) at baseline and post-treatment revealed score reduction in 23 out of 24 (95.8%) V5 recipients (from mean/median 2.2/3 to 0.3/0; P = 6E-010) but only in 1 out of 10 (10%) patients on placebo (1.9/1.5 vs. 1.8/1; P = 0.34). No adverse effects or TB reactivation were seen at any time during follow-up. V5 is safe as an immune adjunct to chemotherapeutic management of TB and can shorten substantially the duration of treatment.

## Introduction

Tuberculosis (TB) is a re-emerging global public health problem, especially in developing countries. Ukraine is a representative country as it concerns the TB epidemic. In 1961, the incidence of TB in Ukraine was 155 cases per 100,000 individuals, which then declined to 32 cases per 100,000 in 1991. However this trend reversed and by 2008 the incidence became 81 per 100,000. The mortality doubled from 10.2/100,000 to 21.6/100,000 between 1990 and 2001 [1]. In addition the Ukraine experiences the worsening epidemic of drug resistant TB. Isoniazid and rifampicin resistance, which defines the MDR-TB, has been found in 44% and 32.9% of TB isolates [2]. The success rates of TB therapy in Ukraine are below average when compared with other regions of the world [3]. Despite availability of TB drugs the situation is far from ideal and it is clear that better therapeutic interventions are needed to reverse the current trend.

Oral therapeutic vaccine V5 was originally developed for the management of chronic hepatitis B and C [4-6]. The preparation is derived from pooled blood of HBV- and HCV-positive donors, which following chemical- and heat inactivation was formulated into an oral pill according to proprietary technology developed by us [6]. It is well known that one third of people carry M. tuberculosis without showing symptoms of the disease. Therefore V5 inherently contains circulating M. tuberculosis antigens. During hepatitis C trial in 20 patients who happened to have pulmonary TB with HIV-co-infection, V5 produced mycobacterial clearance in sputum smears of 94.4% of patients within one month [7]. Subsequent, one-month, placebo-controlled trial in 55 patients confirmed the anti-TB property of V5 revealing negative smear conversion in 96.3% of treated subjects [8]. Our study was, thus, aimed to verify these findings in an independent clinical setting at our TB hospital in Kharkov. The advantage of adding V5 to standard ATT was compared to a treatment regimen consisting of ATT and placebo.

## Materials and methods

### Patients

The study involved 18 first-diagnosed TB (1^st^Dx; 52.9%), 6 relapsed TB (RTB; 17.6%), and 10 patients with confirmed multi-drug resistant TB (MDR; 29.4%). The patient population comprised 7 females and 27 males between ages 20-60 years. The V5 arm had 24 individuals with mean ± SD (median) age 40.8 ± 12 (41) years. The mean/median duration of ATT prior to V5 administration was 1.6 ± 2.5/1 months. The 10 TB patients on placebo had mean ± SD (median) age 34.8 ± 3.58 (35.5) years. The mean/median duration of chemotherapy prior to addition of placebo was 0.5 ± 0.7/0 months. All study patients presented with moderate to severe form of pulmonary TB. Most common symptoms were prolonged heavy cough, pain in the chest, high fever, profuse night sweats, fatigue, dyspnea, hemoptysis, and loss of weight and appetite. Active pulmonary tuberculosis was certified by a medical history and clinical findings compatible with tuberculosis, a chest X-ray showing lung involvement, and positive sputum smear for acid-fast bacilli (AFB) and the culture of *M. tuberculosis*. None of patients in the present study had HIV or viral hepatitis. Conduct of the trial has been approved by the internal review board and has ClinicalTrials.gov Identifier: NCT01222338.

### Randomization, clinical endpoints and exclusion/inclusion criteria

Any patient who presented with pulmonary TB was enrolled into this study and randomly allocated into two arms according to computer-generated random numbers table, i.e., simple randomization procedure. Original plan was to evaluate 35 patients, because we had this specific number of patients available at our hospital wards at the time when study was ready to go. Therefore, we have generated random 35 numbers and have assigned apriori, even numbers to V5 and odd numbers to placebo. The random sequence of digits we have obtained had by chance higher proportion of even than odd numbers: i.e., 24 vs 11. In placebo group we ended up with 10 instead of 11 patients because one patient assigned to this group had declined to participate. The primary endpoint of interest in this study was the effect of the therapy on mycobacterial clearance in sputum smear. Secondary endpoints were changes in biochemical and hematology parameters. The difference in baseline lab and biochemistry characteristics between groups was totally random and their time on TB drugs prior to V5 was also a random event. Additional secondary endpoints of interest were effects on body weight and liver size.

### Treatment regimens

All patients received either standard TB therapy consisting of orally administered Izoniazid (H; 300 mg); Rifampicin (R; 600 mg); Pyrazinamide (Z; 2,000 mg); Ethambutol (E; 1200 mg); and intramuscular injection of Streptomycin (S; 1,000 mg) or individualized treatment regimen as decided by our medical staff based on results of drug resistance testing or prior medical history (Tables 1 and 2). The anti-TB drugs were procured through the centralized national supply system of Ukraine. Patients in V5 and placebo arms received in addition to ATT once-daily tablet of V5 or placebo; usually 30 minutes before or after breakfast. The treatment was administered to hospitalized patients for 30 days under directly observed therapy (DOT). V5 is approved in 2008 by the Ministry of Health of Ukraine as an immunomodulating supplement for the management of chronic hepatitis. As we had not known prior to the accidental discovery that V5 may benefit TB patients we have not much information regarding the exact content of M. tuberculosis antigens [7]. A new formulation (V7) specifically designed for TB is now being tested in preclinical studies and which will be specifically characterized in regard to its mycobacterial antigenic content.

**Table 1:** Baseline and outcome characteristics of TB patients receiving V5 in combination with TB drugs for 30 days

No.	Sex	Age	Months on ATT prior to V5	Dx	TB drugs regime	Smear status		Liver size in cm over normal		Erythrocyte sedimentation rate (mm/h)		Lymphocytes (%)		Leukocytes (× 10^9^/L)		Hemoglobin (g/L)		Body weight (kg)		Total bilirubin (μmol/L)		ALT (mM/h/ml)	
						
						before	after	before	after	before	after	before	after	before	after	before	after	before	after	before	after	before	after
1	M	43	0	1^st^Dx	HRZE	3	1	2	0	50	30	18	23	11.7	9.2	129	122	64	65	8.8	8.8	0.4	0.1

2	M	39	0	1^st^Dx	HRZE	3	1	0	1	17	16	28	25	4.4	5.4	142	139	65	66	9.9	8.8	0.2	0.6

3	M	44	1	1^st^Dx	HRZES	1	0	1	0	21	15	19	29	6.1	5.6	127	116	74	78	8.8	9.9	0.6	0.5

4	M	60	3	1^st^Dx	HRZES	3	0	1	0	27	20	29	31	6.1	6	144	122	71	74	8.8	8.8	0.7	0.1

5	F	27	1	1^st^Dx	HRZES	3	2	1	0	58	40	16	22	11	8	101	110	43	49	8.8	8.8	0.7	0.7

6	M	26	3	1^st^Dx	HRZES	3	1	3	1	15	6	30	37	11.6	6	158	140	66	69	8.8	12.1	0.9	1.7

7	M	57	1	1^st^Dx	HRZES	2	0	0	0	32	9	21	34	5.3	5.1	109	123	60	63	12.1	9.9	0.1	0.5

8	M	31	0	1^st^Dx	HRZES	1	0	0	0	36	26	21	29	7.5	6.6	134	136	62	68	8.8	9.9	0.3	0.4

9	M	39	0	1^st^Dx	HRZES	2	1	0	0	50	30	14	17	11.7	9.6	154	144	63	67	8.8	8.8	0.2	0.2

10	M	31	3	1^st^Dx	HRZES	3	0	0	0	37	2	33	46	9.7	4	145	151	64	67	9.9	8.8	0.4	0.4

11	M	59	0	1^st^Dx	HRZES	1	0	1	1	25	24	27	29	6.6	5.2	138	136	61	66	18.1	12.1	0.3	0.5

12	M	26	0	1^st^Dx	HRZES	3	0	3	1	19	9	24	21	5.7	6.0	156	153	70	73	8.8	8.8	1.3	0.7

13	M	51	1	1^st^Dx	HRZES	3	0	1	2	15	6	16	29	14.2	6.9	142	150	81	81	12.1	8.8	0.6	0.9

14	F	46	1	RTB	HRZES	3	0	0	0	17	3	17	21	8.3	7.7	103	110	52	58	8.8	8.8	0.3	0.1

15	F	54	0	RTB	RZEO	3	0	3	1	16	8	24	34	6.1	5.1	143	128	58	59	8.8	8.8	1.2	0.6

16	F	35	1	RTB	HRZEA	3	0	0	0	32	15	18	37	6.7	5.5	142	136	62	67	8.8	8.8	0.2	0.2

17	M	49	1	MDR	HRZEO	3	0	1	1	55	40	18	37	20.4	7.5	138	100	73	75	12.1	9.9	0.8	0.8

18	M	29	9	MDR	HEAOC	3	1	5	3	25	14	11	26	7.9	7.9	129	138	59	62	8.8	8.8	0.5	0.9

19	F	53	2	MDR	ZEPAO	0	0	0	0	19	17	35	31	4.4	5.8	139	167	55	59	14.3	10.4	0.3	0.4

20	M	29	0	MDR	ZEPAO	2	0	1	0	10	8	33	35	10	6.2	147	148	65	69	8.8	8.8	0.6	0.6

21	M	29	4	MDR	ZEPAO	2	0	3	0	25	17	26	37	10.1	6.8	135	133	57	60	9.9	8.8	1.3	0.6

22	M	49	1	MDR	ZEPO	1	0	2	0	24	17	12	26	5.3	4.9	159	137	72	74	14.2	9.9	1	0.6

23	M	52	0	MDR	ZPAO	1	0	3	1	24	18	24	26	5	4.2	124	128	68	73	13.5	8.8	1.4	0.7

24	M	22	1	MDR	CsCiGF	1	0	0	0	23	18	25	27	19.6	7.2	121	131	66	73	8.8	8.8	0.1	0.2

	5/19	Mean = 40.8 ± 12 Median = 41	Mean = 1.6 ± 2.5 Median = 1	1^st^Dx = 13 RTB = 3 MDR = 8		1/23 2.2/3	18/6 0.3/0	1.3 ± 1.4/1	0.5 ± 0.8/0	28 ± 13.4	17 ± 10.4	22.5 ± 6.8	29.5 ± 6.7	9 ± 4.3	6.4 ± 1.4	135.8 ± 15.8	133.3 ± 15.5	63.8 ± 7.9	67.3 ± 7.3	10.4 ± 2.5	9.4 ± 0.99	0.6 ± 0.4	0.54 ± 0.35
						
						P = 0.000027		P = 0.0018		P = 8.3E-007		P = 2.8E-005		P = 0.0024		P = 0.39		P = 2.3E-009		P = 0.03		P = 0.47	

TB drugs used in this arm are abbreviated as follows: Isoniazid (H), Rifampicin (R), Pyrazinamide (Z), Ethambutol (E), Streptomycin (S), Ofloxacin (O), Amikacin (A), Capreomycin (C), Para-aminosalicylic acid (P), Cs (Cycloserine), Cilastatin (Ci), Gatifloxacin (G), Metronidazole (F), Prothionamide (Pt)

**Table 2:** Baseline and outcome characteristics of TB patients receiving placebo in combination with TB drugs for 30 days

No.	Sex	Age	Months on ATT prior to placebo	Dx	TB drugs regime	Smear status		Liver size in cm over normal		Erythrocyte sedimentation rate (mm/h)		Lymphocytes (%)		Leukocytes (× 10^9^/L)		Hemoglobin (g/L)		Body weight (kg)		Total bilirubin (μmol/L)		ALT (mM/h/ml)	
						
						before	after	before	after	before	after	before	after	before	after	before	after	before	after	before	after	before	after
1	M	22	1	1^st^Dx	HRZSE	1	1	1	0	3	6	22	36	6.1	8.7	155	150	74	75	12.1	9.9	0.3	0.3

2	F	35	0	1^st^Dx	HRZSE	3	3	1	0	22	41	8	10	8	14.1	75	80	45	45	8.8	8.8	0.4	0.6

3	M	40	0	1^st^Dx	HRZSE	1	1	0	1	35	32	12	11	11.8	11.1	158	150	62	62	9.9	9.9	0.4	0.6

4	F	30	0	1^st^Dx	HRZSE	1	1	0	1	8	10	21	16	7.2	7.4	126	120	69	69	8.8	14	0.2	0.6

5	M	20	0	1^st^Dx	HRZSE	3	3	0	0	50	35	27	26	9.2	10.9	116	110	64	66	8.8	8.8	0.3	0.2

6	M	53	2	RTB	HRZSE	2	1	2	1	23	27	19	23	5.5	4.6	86	89	84	86	12.1	8.8	0.3	0.1

7	M	42	0	RTB	HRZSE	3	3	1	1	52	49	19	17	10	9.8	113	115	60	61	8.8	8.8	0.1	0.4

8	M	48	0	RTB	HRZSE	3	3	0	0	47	38	40	37	11	10.3	139	129	60	61	8.8	17.2	0.1	0.4

9	M	22	1	MDR	HRZSE	1	1	1	1	31	20	17	13	8.8	7.7	136	146	70	72	8.8	8.8	0.4	0.6

10	M	36	1	MDR	ZAPPtO	1	1	3	3	33	30	27	25	10.1	9.1	107	131	69	69	21	12.1	0.7	1.7

	2/8	Mean = 34.8 ± 3.58 Median = 35.5	Mean = 0.5 ± 0.7 Median = 0			1.9	1.8	0.9 ± 0.31	0.8 ± 0.29	30.4 ± 5.30	28.8 ± 4.27	21.2 ± 8.9	21.4 ± 9.7	8.77 ± 0.65	9.37 ± 0.80	121.1 ± 8.64	122 ± 7.69	65.7 ± 3.25	66.6 ± 3.39	10.79 ± 1.21	10.71 ± 0.90	0.32 ± 0.05	0.55 ± 0.14
						
						P = 0.34		P = 0.68		P = 0.61		P = 0.91		P = 0.43		P = 0.79		P = 0.01		P = 0.96		P = 0.055	

TB drugs used in this arm are abbreviated as follows: Izoniazid (H), Rifampicin (R), Pyrazinamide (Z), Ethambutol (E), Streptomycin (S), Amikacin (A), Para-aminosalicylic acid (P), Prothionamide (Pt), Ofloxacin (O)

### Laboratory evaluation

The quantitative sputum bacillary load as measured by acid-fast bacilli (AFB) smears was conducted at baseline and at 30 days post-treatment. Smears were scored in a blind fashion from 3 to 0 according to the severity of bacterial load. TB drug resistance was determined by readily available commercial kit (Tulip Diagnostics, Goa, India). The activity of alanine transaminase (ALT) was measured according to the procedure described by Reitman and Frankel [9]. Other biochemistry and hematology parameters were evaluated by standard routine techniques at baseline and at one month post-treatment.

### Statistical analysis

The obtained results were analyzed with statistical software STATMOST (Datamost, South Sandy, UT). The baseline quantitative values relative to the end of study values were evaluated by paired or unpaired Student t-test. Other statistical calculations such as determination of standard deviation, mean and median, were performed with the same software. The non-parametric or categorical values of treatment outcomes were compared by Fisher's exact two-tailed test. All statistical analyses were done on intent-to-treat basis, involving the total number of patients without subgrouping them into responders and non-responders. The resulting probability values were considered as significant at P ≤ 0.05.

## Results

### Lack of adverse reactions

**Figure 1:**
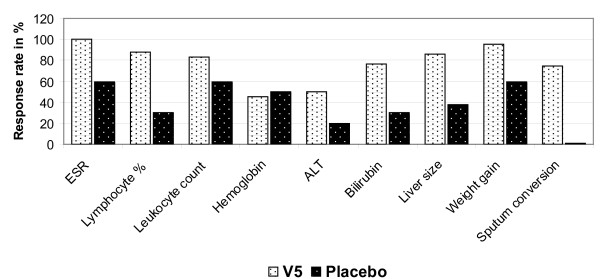
**The proportion of ATT-treated patients in V5 and placebo arms who have benefited from the therapy according to the measured endpoints**.

During the entire duration of follow-up no adverse reactions or reactivation of TB attributable to V5 were identified. Quite contrary patients who were receiving chemotherapy along with V5 fared much better than placebo recipients. While these findings can be considered as subjective, the quantitative endpoints detailed below indicate that the addition of V5 to ATT results in better clinical outcome. The favorable response rates as expressed in percentage values are shown in Figure 1.

### Effect on hematology parameters

The effect of ATT and V5 on select white blood cells and hematology parameters are shown in Tables 1 and 2. Patients in V5 arm displayed positive changes that appeared to be specific to V5 intervention as opposed to the effect of ATT in placebo arm. In particular, the percentage of lymphocytes increased among V5 treated patients (from 22.5 ± 6.8 to 29.5 ± 6.7; P = 2.8E-005) while patients in the control group failed to display any significant changes (21.2 ± 8.9 vs 21.4 ± 9.7; P = 0.91). Elevated leukocyte counts that are traditionally associated with inflammation were reduced in V5-treated patients (9 ± 4.3 vs 6.4 ± 1.4 × 10^9^/L; P = 0.0024) but not in the placebo group (8.77 ± 0.65 vs 9.37 ± 0.80 × 10^9^/L; P = 0.43). Another marker of inflammation, the erythrocyte sedimentation rate (ESR), declined significantly in V5 group (28 ± 13.4 vs 17 ± 10.4 mm/h; P = 8.3E-007) but unchanged in placebo recipients (30.4 ± 5.30 vs 28.8 ± 4.27; P = 0.61). The content of hemoglobin remained at the same level in V5 and placebo patients (135.8 ± 15.8 vs 133.3 ± 15.5 g/L; P = 0.39) (121.1 ± 8.64 vs 122 ± 7.69 g/L; P = 0.79) respectively. In general, V5 appeared to have better effect in normalizing abnormal hematology picture than placebo and ATT (Figure 1).

### Effect on liver size and function

Liver size and serum biochemistry markers of liver function such as ALT and total bilirubin appeared to improve among V5 recipients while patients in placebo group tended to show signs of liver dysfunction (Figure 1). Mean/median liver size in V5 arm reduced from 1.3 ± 1.4/1 to 0.5 ± 0.8/0 cm above normal size (P = 0.002). No changes were seen in placebo recipients (0.9 ± 0.3/1 vs 0.8 ± 0.3/1 cm P = 0.68). While changes in ALT were not statistically significant, the opposite trend in the outcome was observed. ALT levels in V5 patients appeared to decrease from 0.6 ± 0.4 to 0.54 ± 0.35 mM/h/ml (P = 0.47) while in placebo recipients they had almost doubled from baseline values 0.32 ± 0.05 vs 0.55 ± 0.14 mM/h/ml (P = 0.055). Total bilirubin decreased in V5 arm from 10.4 ± 2.5 to 9.4 ± 0.99 μmol/L (P = 0.03) but in placebo no significant decrease was observed 10.8 ± 1.2 vs 10.7 ± 0.9 μmol/L (P = 0.96).

### Effect on body weight

Prior to study initiation patients in both groups had identical underweight problem. The mean baseline weight in V5 and placebo groups was 63.8 ± 8 kg and 65.7 ± 10.3 kg respectively (P = 0.6 by unpaired t-test). The average body weight accrual in V5 group was 3.5 ± 1.8 kg (P = 2.3E-009). Only one patient failed to gain weight, in remaining 23 patients (95.8%) the increase in body mass ranged between 1-7 kg. In placebo arm 60% of patients gained on average 0.9 ± 0.9 kg (P = 0.01), range 1-2 kg.

### Effect on mycobacterial clearance

**Figure 2:**
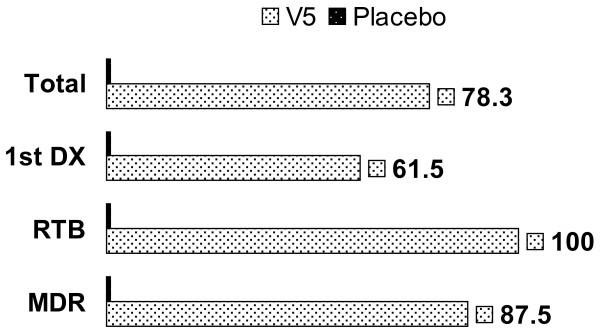
**The percentage of sputum smear-negative patients in placebo and V5 arms administered in combination with TB drugs for one month**. The upper paired bar shows conversion rate in placebo recipients (0%) in comparison to V5 recipients (78.3%) in a total number of enrolled patients (N = 34). Lower paired bars show conversion rates among first-diagnosed TB (1stDx); relapsed TB (RTB); and multidrug-resistant TB (MDR) respectively.

Bacterial clearance was scored in a blinded fashion after AFB staining of sputum smears. One month later 78.3% of V5 patients who had positive sputum smear at baseline had negative findings, while none of patients cleared bacteria in the placebo group - a difference that was statistically significant (P = 0.009 by Fisher's exact two-tailed test) (Figure 2). In placebo group only one patient out of 10 had shown decrease in AFB score (10%) whereas among 24 patients in V5 group everyone (except patient #19) had decreased AFB score (95.8%). Paired t-test of quantitative scoring of sputum bacillary load at baseline and post-treatment revealed that the decrease in V5 group was highly significant (from mean/median 2.2 ± 0.98/3 to 0.29 ± 0.55/0; P = 6E-010) but no difference was seen in placebo recipients (1.9 ± 0.99/1.5 to 1.8 ± 1.03/1; P = 0.34). No differences were seen in conversion rate between 13 first-diagnosed, drug-sensitive TB and 8 multi-drug resistant TB cases in V5 arm (P = 0.62 by Fisher's 2×2 exact test); both forms of TB responded equally well to the immunotherapy (Figure 2).

## Discussion

This placebo-controlled, comparative study involving 34 patients with first-diagnosed TB, relapsed TB, and MDR-TB reveals that when conventional or individualized TB drug regimens are combined with V5, the combination can produce significant improvement in clinical endpoints and clearance of M. tuberculosis at higher rate than in patients on placebo. Biochemistry and hematological analysis of blood samples support favorable adjunctive effect of V5, which has not shown any adverse effects throughout the study duration.

Our findings support two earlier clinical trials of V5 demonstrating favorable outcome in TB patients [7,8]. These studies reported a range of beneficial effects including better quality of life, body weight gain, reversal of ATT-associated hepatotoxicity, reduced inflammation, faster deffervescence, and higher clearance rate of M. tuberculosis in sputum smears. Furthermore, the adjunct immunotherapy resulted in much shorter duration of treatment than among those who received standard ATT. Both prior studies were conducted at the Lisichansk Regional TB Dispensary but these results are now confirmed by the present study performed independently at our TB hospital based in Kharkov.

The normalization of inflammatory indices is considered to have favorable effect on the course of the TB disease [10,11]. There were earlier indications that V5 exhibits the anti-inflammatory activity [4-8]. These observations are supported by this study. Contrary to the outcome in placebo group the markers of inflammation such as elevated leukocyte counts and prolonged erythrocyte sedimentation rate have been significantly reduced in V5 recipients. A favorable change in the blood picture is supported by the increase in total lymphocyte percentage among V5 recipients, but not in the control group. The restoration of suppressed lymphocyte counts and decrease in leukocyte counts is associated with positive treatment outcome [12]. Thus, changes in relative and absolute lymphocyte and leukocyte numbers may hold promise as surrogate markers of treatment response. Further studies aimed at evaluating specific immune phenotypes of peripheral blood cell subsets, including their functional characterization are needed.

The hepatotoxicity induced by anti-TB drugs has serious adverse consequences to treated patients and imposes limitations on treatment options [13]. Addition of V5 appeared to reduce baseline bilirubin and ALT levels, as well as the abnormal liver size when compared to placebo regimen. These observations confirm prior studies in which V5 has been shown to counter the hepatotoxicity of TB drugs [8,9]. For this reason the use of V5 in combination with ATT is advisable to prevent or reverse iatrogenic liver damage.

The immunotherapy has shown clear benefit in reversing body weight loss. The average gain in V5 and placebo groups was 3.5 kg and 0.9 kg which is almost identical to the results of placebo controlled trial involving a comparable group of 55 TB patients from the Lisichansk TB dispensary, i.e., 3.4 kg (59.7 ± 8 vs 63.1 ± 9 kg; P = 5.7E-007) and 1.07 kg (59.1 ± 10 vs 60.1 ± 10.4 kg; P = 0.003) respectively [8]. In contrast, the mean weight gain observed in the earlier conducted, open-label trial involving 20 patients with HIV-TB was 7.7 kg (P = 4.6E-007) [7]. This almost two-fold discrepancy is perhaps due to the fact that all these patients had HIV infection - a condition that is associated with worsened wasting. Nevertheless, there appears to be the strong relationship between body weight gain and favorable outcome, which needs to be confirmed in a larger cohort of patients in independent clinical settings [14,15].

Conversion of sputum smear from positive to negative is a main indicator of the efficacy of anti-TB intervention. We have observed that V5 accelerates and significantly enhances bacillary clearance as compared to control group on ATT (Figs. 1 and 2). This observation supports earlier studies at Lisichansk TB dispensary which reported similar results albeit at higher conversion rate [7,8]. The difference in outcome between placebo and V5 recipients was also significant. In the prior placebo-controlled study the conversion in placebo arm was seen in 25% of patients while in our hands 0% converted. We do not know what the reason for such a discrepancy is. In Arjanova et al., study the duration of ATT prior to study initiation 4 ± 3/3 and 3.4 ± 2.7/3 months respectively [8]. In our study mean ± SD/median duration of ATT prior to V5 and placebo administration was shorter and uneven 1.6 ± 2.5/1 versus 0.5 ± 0.7/0 months. This discrepancy was mainly due to the presence of outlier patients in V5 group. Particularly, five patients #4, #6, #10, #18, and #21 were on TB drugs for 3, 3, 3, 9, and 4 months respectively (Table 1). Nevertheless, while difference in time on TB drugs can account to some degree for discrepancy in obtained results, we do not think that this was the main reason. This is supported by stratified analysis of patients on V5. If we exclude outlier patients we have 19 individuals who were on TB drugs for an average 0.57 months, which is same as in the placebo group (P = 0.76; by unpaired t-test). In this subgroup of patients 15 out of 19 (78.9%) patients had converted after one month, which is identical to the original conversion rate 78.3% we have observed for V5 group as a whole. Thus, while longer pre-treatment with TB drugs may contribute to higher conversion rate as shown by Lisichansk study, in our case this contribution was negligible, since the conversion rate was still drastically different between V5 and placebo, regardless how long they were treated with TB drugs. Nevertheless, additional studies are needed to assess the effect of the length of prior exposure to ATT in relation to the conversion rate induced by immunotherapy.

V5 contains heat-inactivated M. tuberculosis antigens which are commonly present in the blood of people who do not have active TB disease [16]. In this sense V5 resembles therapeutic vaccine RUTI which contains detoxified M. tuberculosis antigens [17]. There are several other immune adjuncts of bacterial origin which enhance sputum conversion when used together with ATT. These include M. vaccae [18], M. phlei [19], M. w [20], and Likopid [21]. Even attenuated M. bovis preparation, known as BCG vaccine since 1921, has been occasionally used as a therapeutic modality. For example, in Chinese army trial involving 360 patients with MDR-TB the negative sputum conversion rate in BCG recipients was 98.3% and 97.2% in chemotherapy control. While this difference was not significant the recurrence of TB after 5 years was 2.3% in the BCG group, but 6.9% in the control group [22]. The authors of this study, however, did not mention whether use of BCG was accompanied by reactivation of TB or aggravation of disease symptoms - a phenomenon first observed by Robert Koch - the discoverer of Mycobacterium tuberculosis.

Koch's phenomenon of TB reactivation has been thwarting the development of safe and effective therapeutic vaccine since 1890 [23]. While the risk of inducing deleterious Koch-like reaction appears to be relatively low in humans [24], extreme caution is still needed as it occurs commonly in animal models of post-exposure vaccination when various TB vaccine candidates, including BCG, were tested and produced occasionally lethal outcomes [25-30]. Recent BCG revaccination trial in the area of high TB endemicity with increased probability of occult disease has produced worrisome outcome, which might be interpreted as a fatal Koch-like TB reactivation. The trial carried out in Guinea-Bissau on 2871 children was stopped prematurely because of a cluster of deaths in the BCG arm of the study [31]. In our case we have not seen any reactivation of the disease; quite contrary we have consistently observed the reduction of inflammation signs such as ESR, leukocytosis and fever. This, we believe, is largely due to the well-known fact that orally delivered antigens induce immune tolerance instead of immune activation, e.g., Koch's reaction [32]. An immunomodulator we have studied extensively in the past is a multiherbal extract Dzherelo (Immunoxel) which has shown similar properties as V5 on the conversion rate in drug-sensitive as well as MDR-TB and reduction of inflammation [11,33]. Contrary to TB drugs the efficacy of immune-based therapy on drug-resistant TB is considered to be the same as against drug-sensitive strains [34]. In our hands V5 seemed to be equally effective against both forms of TB, as no statistical difference was observed when we compared the conversion outcome between first-diagnosed TB and relapsed/MDR-TB subsets of patients.

## Conclusions

V5 is shown to be safe and capable of reversing ATT-associated hepatotoxicity. In addition, V5 reduces the inflammation as evidenced by several hematological and biochemical markers. Weight gain and sputum smear conversion rate have been significantly enhanced as compared to conventional ATT. As evidenced by obtained improvements the combination of V5 and ATT can shorten substantially the duration of treatment. Our preliminary findings need to be explored further in a larger population of patients followed for longer periods of time to establish the usefulness of V5 as a therapeutic TB vaccine.

Considering that 2 billion people are latently infected with M. tuberculosis, it is likely that two different types of prophylactic TB vaccines will be needed: one is so called pre-exposure vaccine to prevent mycobacterial infection in naïve individuals and second, post-exposure vaccine, to prevent TB disease in tubercle bacilli carriers without exacerbating disease manifestations. Up to now, the majority of vaccine candidates are in the first category and only M. vaccae and RUTI are considered to be in the second category [35]. Studying V5 as post-exposure vaccine can contribute to better understanding of the immunopathogenesis of TB resulting in the design of effective vaccines and accelerate evaluation of their efficacy. In addition, V5 studies can yield important insights into correlates of immune protection, which are still poorly understood [36].

## List of abbreviations

1^st^Dx: first-diagnosed; A: amikacin; AFB: acid-fast bacilli; ALT: alanine transaminase; ATT: anti-tuberculosis therapy; BCG: Bacille Calmette-Guérin; C: capreomycin; Ci: cilastatin; Cs: cycloserine; DOT: directly observed therapy; Dx: diagnosis; ESR: erythrocyte sedimentation rate; F: metronidazole; G: gatifloxacin; H: izoniazid; Hb: hemoglobin; HBV: hepatitis B virus; HCV: hepatitis C virus; HIV: human immunodeficiency virus; MDR: multi-drug-resistant; O: ofloxacin; P: para-aminosalicylic acid; Pt: prothionamide; R: rifampicin; RTB: relapsed TB; S: streptomycin; SD: standard deviation; TB: tuberculosis; V5: Immunitor V-5; Z: pyrazinamide.

## Competing interests

ASB and VJ are officers and owners of Immunitor company, which manufactures V5. All other authors declare that they have no competing financial interests.

## Authors' contributions

DAB and YNP carried out the planning and conduct of the study, data collection and manuscript preparation. ALS, AIC and TSB took part in the conduct of study, data collection and interpretation. DB and ASB participated in editing of the manuscript and statistical analysis of data. VJ provided samples of V5 and preparations of the placebo and provided guidelines for conduct of the study. SIZ supervised medical personnel and arranged the ethical approval of the study. All authors read and approved the manuscript.
